# Optimising Nurse–Patient Assignments: The Impact of Machine Learning Model on Care Dynamics—Discursive Paper

**DOI:** 10.1002/nop2.70195

**Published:** 2025-04-23

**Authors:** Mutaz I. Othman, Abdulqadir J. Nashwan, Ahmad A. Abujaber

**Affiliations:** ^1^ Nursing Department Hamad Medical Corporation Doha Qatar

**Keywords:** health care, machine learning, nurse competencies, nurse–patient assignment, patient outcome

## Abstract

**Background:**

Machine learning (ML) models can enhance patient–nurse assignments in healthcare organisations by learning from real data and identifying key capabilities. Nurses must develop innovative ideas for adapting to the dynamic environment, managing staffing and establishing flexible workforce solutions.

**Aim:**

This discursive paper discusses the application of ML in optimising patient–nurse assignments within healthcare settings, considering various factors such as staff skill mix, patient acuity, cultural competencies and language considerations.

**Methods:**

A discursive approach was used to optimise nurse–patient assignments and the impact of ML models. Through a review of traditional and emerging perspectives, factors such as staff skill mix, patient acuity, cultural competencies and language‐related challenges were emphasised.

**Results:**

Machine learning models can potentially enhance healthcare patient–nurse assignments by considering skill integration, acuity level assessment and cultural and language barrier awareness. Thus, models have the potential to optimise patient care through dynamic adjustments.

**Conclusion:**

The application of ML models in optimising patient–nurse assignments presents significant opportunities for improving healthcare delivery. Future research should focus on refining algorithms, ensuring real‐time adaptability, addressing ethical considerations, evaluating long‐term patient outcomes, fostering cooperative systems, and integrating relevant data and policies within the healthcare framework.

No patient or public contribution.

## 1 Introduction

Patient–nurse assignments in healthcare organisations are crucial for several reasons. Firstly, appropriate nursing staffing facilitates an effective match between the requirements of patients and their families and the competencies of nurses, which is essential for patient safety and quality of care (Halm 2019). Nurses ensure timely coordination and communication of the patient's condition (Cho et al. 2016). Secondly, appropriate nursing staffing contributes to higher nurse satisfaction, which can improve patient care and outcomes (Griffiths et al. 2016). Appropriate nursing staffing can decrease care costs as fewer patients require additional resources and interventions (Dall'Ora et al. 2022). appropriate nurse staffing also affects the ability of nurses to work in interdisciplinary partnerships with other healthcare professionals, ensuring comprehensive and coordinated patient care (Nashwan et al. 2023; West et al. 2014). The staffing principles should be dynamic and adaptable to the ever‐evolving nature of health care, allowing for adjustments based on changing patient needs and healthcare environments (Atiemo 2023). Lastly, healthcare organisations should have well‐developed staffing guidelines with measurable nurse‐sensitive outcomes to guide daily staffing decisions (Halverson and Scott Tilley 2022).

Healthcare organisations can use machine learning (ML) models to facilitate staff unity. These models can acquire skills from real data by applying ML algorithms. By identifying the essential competencies and experiences of staff nurses in the staffing pool and aligning them with patient care requirements, these models can also mitigate safety incidents in healthcare organisations and enhance staff capacities (Qayyum et al. 2020). Healthcare organisations must prioritise the development of practical staff nurse skills and subject matter expertise to incorporate AI into their workforce successfully and result in positive business outcomes (Sofia et al. 2023). The deployment and management of ML models require the expertise of ML engineers, who work closely with data scientists to put models into production. Overall, developing successful ML projects is a collaborative job requiring various expertise (Sofia et al. 2023).

This discursive analysis focuses on optimising patient–nurse assignments in healthcare settings through ML methods. The factors that must be considered in patient care include staff skill mix, patient acuity levels, cultural competencies and language‐related considerations (Griffiths et al. 2014; Sliwinski et al. 2024).

## 2 Patient–Nurse Assignments

The traditional approach to patient–nurse assignments in healthcare organisations involves assigning nurses based on room proximity and mandated nurse‐to‐patient ratios (Meyers 2019). This approach may only sometimes consider the intensity of nursing care required for each patient and can lead to imbalances in workload and staffing levels. It also focuses on completing tasks rather than providing personalised care tailored to each patient's needs (Larson et al. 2017). Additionally, the individual method of assigning nurses can result in consistency in care delivery and outcome evaluation. The challenges associated with manual processes in patient–nurse assignment include imbalances in workload and an uneven distribution of nursing effort due to variations in educational activities, interventions and psychosocial needs (Jiang et al. 2023).

Manual assignment systems may lack adaptability to changes in patient‐related tasks, such as psychosocial status, medical status, care transitions and nursing care plans (Forton 2018; Tomic 2017). Also, challenges in managing skill diversity, the traditional method of assigning nurses according to their abilities and expertise can result in an unequal distribution of workload and staffing (Al‐Dweik and Ahmad 2019). Furthermore, the task‐oriented approach prioritises task completion over providing individualised care that meets each patient's specific needs, leading to a fragmented approach to patient care (Parreira et al. 2021). To address these limitations, healthcare organisations are exploring alternative approaches, such as ML models and workload management tools, to optimise staffing levels and improve patient care (Ahmed et al. 2020).

Nurse–patient interaction in patient–nurse assignments is critical in improving care outcomes and patient satisfaction by understanding cultural, religious and linguistic preferences (Evans 2016). Cultural competence is a continuous process where nurses work effectively within a patient's cultural context, ensuring fair healthcare access and opportunities (Sindayigaya 2017). Cultural competence involves understanding the patient's uniqueness, beliefs and expectations, providing patient‐centred care, empathy, advocacy and respect (Douglas et al. 2014). Culturally competent nursing care is guided by four principles: care is designed for the specific patient, based on the uniqueness of the person's culture, includes self‐empowerment strategies and provides sensitivity based on the cultural uniqueness of patients (Jackson 2016; Sindayigaya 2017). Culturally competent healthcare leads to better education, increased healthcare‐seeking behaviour, appropriate testing, fewer diagnostic errors, improved adherence to medical advice and greater access to quality clinicians (Alghazali 2023). Therefore, integrating staff skills, including cultural competency, is essential to ensure efficient assignments (Okere 2022). Integrating staff competencies is critical for optimising patient–nurse assignments by overcoming the difficulties encountered in manual assignment systems (Al‐Dweik and Ahmad 2019). So automated data nursing assignment tools that integrate workload acuity scores and conventional nurse‐to‐patient ratios can aid in managing nursing workload and adjusting to shifts in patient requirements (Meyer et al. 2020).

## 3 Method

This paper explores the impact of ML models on patient–nurse assignment in healthcare settings. A discursive approach examines complex factors and critically analyses diverse perspectives. A broad review of existing literature was conducted to understand both traditional and emerging perspectives on nurse–patient assignments and the role of ML in health care. To guide the discursive analysis, this paper organises the discussion around the four central and pivotal factors that influence patient–nurse assignments: skill mix, patient acuity level, cultural competence and language barriers (Duffy 2016), as described in Table 1. Due to its discursive nature, this paper does not require the Research Ethics Committee's approval. The paper critically examines how ML could enhance or disrupt existing practices, using examples from current implementations in healthcare settings.

**TABLE 1:** Description of factors that must be considered in patient–nurse assignments.

Skill mix	Patient acuity	Cultural competences	Language‐related issues
The nursing skill mix is a multifaceted concept that includes the quantity, expertise and educational qualifications of nurses working in healthcare settings (Aiken et al. 2017)	Patient acuity is a metric used by charge nurses to assign nurses based on patient demand properly, ensuring workload balance and optimising patient safety and care quality (Brennan et al. 2019)	Nurses' cultural competency refers to their capacity to provide safe and high‐quality health care to patients from various cultural backgrounds. It requires cultural awareness, sensitivity, knowledge, skills and ongoing development (Sharifi et al. 2019). Antecedents encompass cultural diversity, interactions and a curiosity for understanding various cultures. Cultural competence results in advantages for patients, nurses and healthcare organisations	Language‐related issues refer to challenges and barriers to language differences between individuals, particularly in healthcare settings. These issues can include difficulties in communication, misunderstandings, a lack of trust and inadequate information provision. In the context provided, language‐related issues were observed in nurses' efforts to communicate with patients from diverse cultural backgrounds (Lyytikäinen and Tran 2017)

To meet these requirements, nurses must develop innovative ideas that can readily adapt to the dynamic healthcare environment, considering the diverse staffing approaches used in hospitals and establishing adaptable workforce solutions. Managing staffing is essential for registered nurses to effectively contribute to establishing staffing arrangements. This involves close collaboration with other healthcare professionals to ensure adequate time is allocated to patients, meeting their care needs and fostering collaborative interdisciplinary partnerships (Yoder‐Wise and Sportsman 2022). By prioritising the integration of staff expertise and workload management processes, healthcare organisations may enhance staffing efficiency and the quality of patient care (Dawson et al. 2014). This ensures that nurses can deliver tailored, personalised treatment that meets each patient's needs.

## 4 Results

**FIGURE 1:**
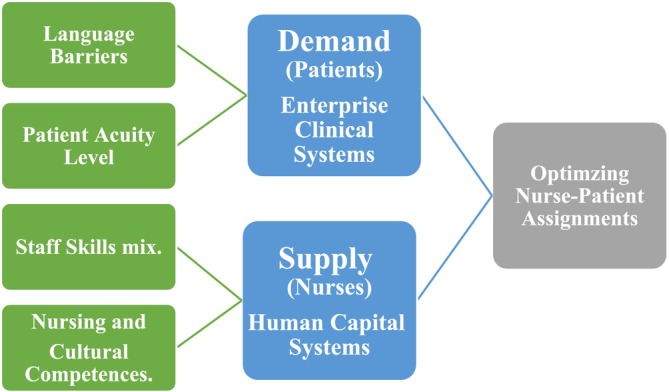
Flowchart overview of the optimising patient–nurse assignment process.

This paper focuses on four key considerations for optimising nurse–patient assignment, as illustrated in Figure 1. The argument will focus on the factors shaping nurse–patient assignment.

### 4.1 Patient–Nurse Assignments and Skill Mix

The critical role of patient–nurse assignments and skill mix influences positive outcomes for patients, nurses and the overall healthcare environment (Havaei 2016). Several nursing skill mix models are under investigation, highlighting the significance of adequately trained and equipped nurses (Powell et al. 2016). The complex relationship between patient–nurse assignments and skill mix highlights the need for a clear vision and the inclusion of various healthcare professionals, such as nurse practitioners and physician assistants. A multifactorial skill‐matching approach in intensive care units is crucial for ensuring patient safety, and the assignment process is influenced by multiple factors (Griffiths et al. 2023; Powell et al. 2016). The significance of a deliberate and strategic approach to patient–nurse assignments and skill mix is emphasised, focusing on patient safety, care quality and staff well‐being.

### 4.2 Patient–Nurse Assignments and Patient Acuity

Considering patient acuity when assigning nurses to patients has been consistently emphasised. High‐acuity patients can lead to nurse fatigue and increased workload, highlighting the importance of balancing assignments with lower‐acuity patients (Al‐Ruzzieh et al. 2023). The importance of evidence‐based leadership strategies in addressing turnover and staffing issues related to patient care is highlighted (Li et al. 2018), whereas Eastman and Kernan (2022) created a patient acuity tool to facilitate fair assignments, resulting in enhanced staff satisfaction. These findings emphasise the significance of a thorough approach that accounts for patient acuity and nurses' workload in nurse–patient assignments. The study also looks at how to use patient classification systems, problems when hiring staff and optimisation models that use patient acuity indicators and nursing workload scores to ensure that everyone is assigned the right amount of work and that no one feels too overwhelmed.

### 4.3 Patient–Nurse Assignments and Cultural Competences

Understanding cultural differences is crucial in patient–nurse assignments, particularly when patients' cultural beliefs conflict with medical advice (Sindayigaya 2017). Nurses should perform culturally sensitive assessments and integrate diverse needs into patient‐centred care plans. Nurses face challenges, such as diverse patient populations, limited resources and biases, despite the significance of cultural competence (Narayan and Mallinson 2022). Nursing education, research and policy development strategies must address these challenges (Nashwan 2024). Understanding nurses' perspectives on challenges and barriers is crucial for improving care delivery practices and providing effective patient‐centred care.

### 4.4 Patient–Nurse Assignments and Language‐Related Issues

Language barriers between nurses and patients negatively impact the quality of care, leading to misunderstandings that disrupt patient engagement and compromise safety (Gerchow et al. 2021). Nurses encounter obstacles in addressing these barriers, including using interpreters and the requirement for cultural proficiency. The growing cultural diversity among nurses and patients adds complexity to communication, underscoring the significance of comprehending intercultural nurse–patient communication (Lyytikäinen and Tran 2017). Additionally, addressing language barriers in the healthcare system is crucial for improving safety and patient satisfaction.

## 5 Discussion

### 5.1 Machine Learning Models

Machine learning is artificial intelligence that allows systems to learn from data and identify patterns without human intervention. By improving patient care and optimising data management, ML has played a vital role in health care (Ahmed et al. 2020). This system allows healthcare practitioners to gather and analyse patient data, detect healthcare trends and recommend patient treatments. In addition to improving decision‐making, mitigating risks and enhancing patient health outcomes, ML can benefit decision‐making (Dash et al. 2019).

Machine learning models can be employed in patient–nurse assignments to enhance staffing efficiency, balance the nursing workload and adjust to fluctuations in patient requirements (Schäfer et al. 2023). To provide more precise and practical assignments, these models can consider multiple aspects, including patient acuity, nurse competencies and patient preferences. Machine learning models can automate the assignment process, ensuring each patient is paired with the most suitable nurse (Schäfer et al. 2023). This can result in improved outcomes and increased patient satisfaction.

Healthcare organisations can benefit by incorporating staff knowledge and skills through ML models. These models facilitate the identification of the abilities and knowledge possessed by each registered nurse, enabling a more precise and efficient allocation of tasks and responsibilities (Wang et al. 2018). By incorporating staff expertise, healthcare organisations can guarantee appropriate personnel allocation for specific tasks, resulting in increased efficiency, enhanced results and heightened patient satisfaction. Moreover, ML models can assist in identifying the specific training and development requirements, enabling organisations to allocate resources towards improving the abilities that will have the most significant effects on patient care and overall organisational performance.

Implementing ML in patient–nurse assignments may pose several challenges that must be addressed. Healthcare professionals may face difficulties familiarising themselves with the new system, requiring training programmes and effective communication strategies to ensure a smooth transition. Ongoing training is essential as ML systems evolve, and technical issues such as software bugs or compatibility problems can hinder system effectiveness. Additionally, the slowness of the system and system downtime, whether planned or unplanned, can impact usability and patient care. Proactive measures such as continuous education, regular maintenance and quick response protocols are crucial to overcoming these challenges and maximising the benefits of ML in patient–nurse assignments.

### 5.2 Impact of ML Models on Patient–Nurse Assignments

Machine learning models have the potential to revolutionise patient–nurse assignments in healthcare settings. By analysing nurse competencies and patient acuity levels, these models can make more accurate and precise assignments (Kumar et al. 2023). Each patient is matched with the most suitable nurse, considering their unique skills and experience. As a result, patient care and satisfaction are greatly enhanced.

Furthermore, ML models can also consider cultural competence and language barriers in patient care. This ensures patients receive care sensitive to their cultural and linguistic needs (Hilty et al. 2020). These ML models foster equitable and culturally appropriate task assignments by analysing data points such as patient preferences and nurse competencies.

In addition to improving patient care, ML models can positively impact cost reduction and efficiency (Al‐Jarrah et al. 2015). By optimising patient–nurse assignments and integrating staff skills, these models can reduce the length of hospital stays, prevent readmissions and improve overall patient outcomes. Hospitals can achieve cost savings and allocate resources more efficiently by assigning the most appropriate nurse to each patient and distributing tasks fairly. Overall, employing ML models has the potential to influence patient–nurse assignments greatly.

### 5.3 Guiding Patient–Nurse Assignments Through ML Models

ML models can analyse extensive amounts of data to detect patterns and trends, enabling more precise predictions of patient requirements and the selection of the most suitable nurse to address those needs. By carefully considering these factors, ML models can ensure that every patient is paired with the most appropriate nurse (Johnson et al. 2016). This, in turn, leads to more equitable workloads and enhances the quality of patient care.

Machine learning algorithms can be utilised within electronic staffing systems such as Cerner Clairvia to track patient data and assess various factors contributing to the nursing workload, including dynamic patient events. These events can lead to sudden changes in workflow within a hospital setting, impacting staffing needs. By leveraging ML, hospitals can better predict and adjust staffing targets in response to these dynamic patient events, ensuring optimal nursing performance (Borowski 2013).

### 5.4 Analysing Patient Outcomes Through Nurse Assignments

Emphasises the transformational potential of ML models in health care, particularly for predicting patient outcomes through nurse assignments. By systematically analysing historical data, these advanced models reveal detailed patterns that can significantly improve our knowledge of the dynamics impacting patient care, as well as enhance their decision‐making process regarding patient–nurse assignments. So, ML models consider a variety of factors, including the nurse's skills and experience, the patient's medical history and preferences, and special care needs (Goodwin et al. 2003).

Machine learning models can enhance accuracy, consider multiple factors and predict patient outcomes based on nurse assignments. Healthcare organisations can use these models to enhance patient–nurse assignments, improving patient care and outcomes.

**FIGURE 2:**
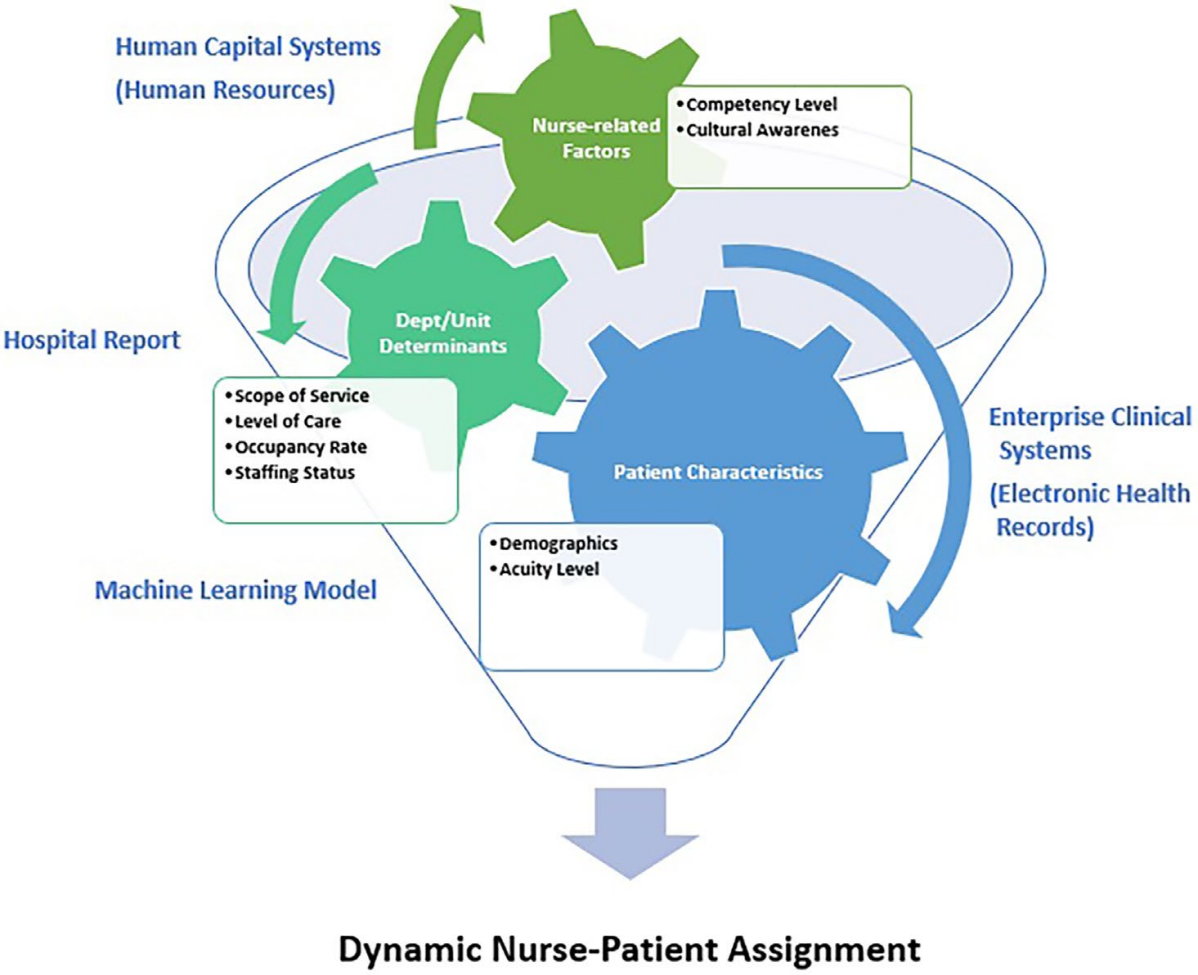
Machine learning–guided patient–nurse assignment process.

The process of patient–nurse assignments, as shown in Figure 2, is organised into distinct categories, starting with data collection. This involves gathering information from patients and nurses and assessing patient acuity levels. Efficiently processing the collected data requires a skill mix, matching competencies, predicting acuity levels, analysing cultural aspects, considering language barriers and ensuring equitable task assignments. The next step is optimising assignments by matching patients with suitable nurses and making dynamic adjustments for optimal care. This mind map offers a clear and scholarly overview of how ML models facilitate patient–nurse assignments. It demonstrates the integration of staff skills, competencies, patient acuity levels, cultural considerations, equitable task assignments and language barriers in patient care.

## 6 Conclusion

In conclusion, patient–nurse assignments in healthcare organisations have traditionally been based on proximity and mandated ratios, leading to imbalances in workload and a focus on task completion rather than personalised care. However, healthcare organisations are now exploring alternative approaches, such as ML models and workload management tools, to optimise staffing levels and improve patient care. By integrating staff skills and using automated data nursing assignment tools, healthcare organisations can overcome the challenges of manual assignment processes and ensure efficient and personalised care for each patient.

Machine learning models could revolutionise patient–nurse assignments by considering nurse competencies, patient acuity levels, cultural competence and language barriers. Future research should focus on refining algorithms, examining real‐time adaptation, addressing ethical and cultural considerations, assessing long‐term patient outcomes, developing collaborative systems, integrating additional data sources and informing healthcare policies using ML models. This will advance the field of ML‐guided patient–nurse assignments and explore further contributing factors to nursing assignments. Furthermore, this would improve patient satisfaction, clinical outcomes, costs and operational efficiency. Overall, ML models have the potential to significantly influence patient–nurse assignments and the integration of staff skills in health care, ultimately enhancing the quality of patient care.

## Author Contributions

M.I.O., A.J.N. and A.A.A. made substantial contributions to the conception and design, or acquisition of data, or analysis and interpretation of data. M.I.O., A.J.N. and A.A.A. involved in drafting the manuscript or revising it critically for important intellectual content. M.I.O., A.J.N. and A.A.A. given final approval of the version to be published. Each author should have participated sufficiently in the work to take public responsibility for appropriate portions of the content. M.I.O. agreed to be accountable for all aspects of the work in ensuring that questions related to the accuracy or integrity of any part of the work are appropriately investigated and resolved.

## Conflicts of Interest

The authors declare no conflicts of interest.

## Data Availability

Data sharing is not applicable to this article as no new data were created or analysed in this study.
